# Bladder-Sparing Chemoradiotherapy Combined with Immune Checkpoint Inhibition for Locally Advanced Urothelial Bladder Cancer—A Review

**DOI:** 10.3390/cancers14010038

**Published:** 2021-12-22

**Authors:** Jons W. van Hattum, Ben-Max de Ruiter, Jorg R. Oddens, Maarten C. C. M. Hulshof, Theo M. de Reijke, Adriaan D. Bins

**Affiliations:** 1 Department of Urology, Amsterdam University Medical Centers, University of Amsterdam, Cancer Center Amsterdam, 1081 HV Amsterdam, The Netherlands; j.w.vanhattum@amsterdamumc.nl (J.W.v.H.); b.deruiter@amsterdamumc.nl (B.-M.d.R.); j.r.oddens@amsterdamumc.nl (J.R.O.); t.m.dereyke@amsterdamumc.nl (T.M.d.R.); 2 Department of Radiation Oncology, Amsterdam University Medical Centers, University of Amsterdam, Cancer Center Amsterdam, 1081 HV Amsterdam, The Netherlands; m.c.hulshof@amsterdamumc.nl; 3 Department of Medical Oncology, Amsterdam University Medical Centers, University of Amsterdam, Cancer Center Amsterdam, 1081 HV Amsterdam, The Netherlands

**Keywords:** bladder cancer, bladder-sparing treatment, immune checkpoint inhibitors

## Abstract

**Simple Summary:**

Immunotherapy with immune checkpoint inhibition (ICI) has provided durable treatment responses in advanced, metastatic, bladder cancer patients. The first trials using checkpoint inhibitors before surgery, when the cancer is still confined to the pelvis, without signs of metastasis, have reported promising results. We reviewed the literature to identify clinical trials combining ICI with bladder-sparing chemoradiotherapy (CRT). Radiotherapy stimulates the immune system, thereby possibly inducing an additive effect in combination with checkpoint inhibition. Currently, twelve trials are treating patients with this immunochemoradiotherapy (iCRT) combination treatment. Several combinations with different chemotherapeutics and ICI added to CRT appear safe and feasible. Further research and comparative trials are needed to prove whether iCRT has additional clinical value for bladder cancer patients.

**Abstract:**

Despite current treatment strategies, the 5-year overall survival of muscle-invasive bladder cancer (MIBC) is approximately 50%. Historically, radical cystectomy (RC) with neoadjuvant chemotherapy has been the first-choice treatment for this patient group. Recently, several studies have reported encouraging results of using immune checkpoint inhibitors (ICI) prior to RC. However, in recent years, bladder-sparing alternatives such as CRT have gained popularity. The effect of radiotherapy on the tumor microenvironment is an important rationale for combining CRT with ICI therapy. Worldwide, twelve immunochemoradiotherapy (iCRT) trials are ongoing. Each study employs a different chemotherapy and radiotherapy regimen and varies the timing of ICI administration concurrent to radiotherapy, adjuvant, or both. Five studies have presented (preliminary) results showing promising safety and short-term survival data. The first peer-reviewed publications are expected in the near future. The preclinical evidence and preliminary patient data demonstrate the potential of iCRT bladder-sparing treatment for bladder cancer.

## 1. Introduction

Urinary bladder cancer (UBC) ranks among the top 10 most prevalent cancers worldwide [1]. Histologically, it consists predominantly of urothelial carcinoma; however, despite advances in cancer care leading to increased survival for most cancer types, the prognosis for UBC patients has not improved significantly in recent decades [2].

In fact, UBC that invades the detrusor muscle (MIBC) has a poor prognosis with a 5-year overall survival (OS) of approximately 50% [3,4].

Since the 1970s, treatment guidelines have advised RC with bilateral pelvic lymph node dissection as the preferred treatment option for MIBC [5]. Neoadjuvant platinum-based chemotherapy (CT) has shown a modest survival benefit of 5–8% on the 5-year OS [6,7]. 

The recent introduction of immune checkpoint inhibitors (ICI) has changed the field of oncology [8]. In metastatic urothelial carcinoma (mUC), five ICI have been extensively studied. These ICI target cytotoxic T lymphocyte antigen 4 (CTLA-4), programmed cell death 1 receptor (PD-1), or its ligand (PD-L1) and have shown durable responses, combined with a favorable toxicity profile, compared to platinum-based chemotherapy [9]. Patients with tumors that express high levels of PD-1 or PD-L1 in the tumor appear to benefit most from this therapy [10,11,12].

Based on these results, several trials have been conducted for nonmetastatic UBC patients who are ineligible for cisplatin-based CT as a neoadjuvant treatment before RC. The results of the PURE01 and ABACUS trials have confirmed the feasibility of ICI monotherapy as induction therapy before RC [13,14]. Additionally, two studies have reported promising results with pathological complete response (pCR) rates of 33–45% for anti-CTLA-4/anti-PD-(L)1 combination treatment before RC [15,16]. Although as of yet no phase 3 results are available, these results have led to further exploration of incorporating ICI into curative MIBC treatment strategies. In addition to RC, one of these potential treatment strategies is chemoradiotherapy (CRT).

Bladder-sparing treatment modalities such as radiation therapy (RT) have historically been considered inferior to RC. In recent years, radiotherapy has gained popularity due to several developments. Firstly, improved treatment planning, and thereby reduction of radiation damage of healthy tissue, has contributed to reduced side effects [17]. Secondly, radiosensitization with mitomycin C + fluorouracil has significantly improved treatment outcomes in MIBC patients [18]. Therefore, CRT is currently the preferred bladder-sparing treatment over RT alone [4]. Although RC is still considered the primary treatment option for MIBC, direct comparative trials between RC and CRT are lacking. Studies using indirect comparative measures such as propensity score-matching or meta-analyses did not show a survival benefit for RC compared to CRT [19,20]. Altogether, CRT may be more broadly applicable in MIBC care. 

**Figure 1:**
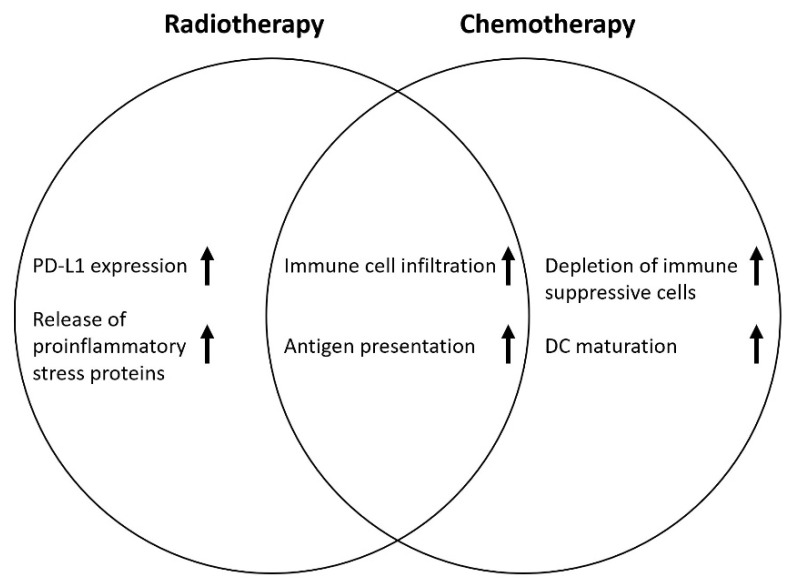
Proposed synergistic effects on the TME of RT and CT on ICI treatment. DC: dendritic cell maturation.

The cytotoxic effect of radiotherapy results from the DNA damage mediated by ionizing radiation leading to cancer cell apoptosis. In preclinical research, multiple effects of RT on the tumor microenvironment (TME) have been reported, such as remodeling of the vasculature, influencing lymphocyte extravasation, increasing tumor-infiltrating lymphocytes and antigen-presenting cells, and the release of stress proteins such as HMGB1 and ATP [21]. Inversely, RT has the potential to increase the effect of ICI. Several preclinical and phase 1 studies have reported synergistic effects of RT and ICI, e.g., through upregulation of PD-L1 expression and cross-presentation of tumor antigens [22,23]. Likewise, CT has the potential to enhance the antitumor effect of ICI, thereby priming the TME following CRT (Figure 1) [24,25].

In the first phase 3 trial reporting on the combination of CRT and ICI, patients with stage III non-small-cell lung cancer (NSCLC) were treated with CRT and sequentially received the PD-L1 inhibitor durvalumab or placebo. Anti-PD-L1 treatment resulted in prolonged progression-free survival (PFS) and overall survival (OS) compared to placebo [26]. These findings support the rationale for combining CRT with ICI in MIBC.

There are several reviews summarizing the current early evidence on ICI therapy before or after RC [27,28,29]. However, reviews on ICI combined with bladder-sparing CRT for MIBC are lacking. Given the trend of ICI implementation in the curative setting of cancer and the broader use of organ-sparing treatments such as CRT, this review provides an overview of the existing evidence on combining ICI with CRT (iCRT) in nonmetastatic MIBC.

## 2. Clinical Evidence

We performed a systematic search throughout October 2021 in MEDLINE, EMBASE, the Cochrane Library, and clinicaltrials.gov using the keywords ‘muscle-invasive bladder carcinoma’, ‘immune checkpoint inhibition’, ‘radiotherapy’, ‘chemotherapy’, and synonyms. A total of 1993 articles were screened on title and abstract using Rayyan [30] by two authors (J.H. and B.R.).

Thus far, one study published peer-reviewed iCRT results for MIBC [31]. Currently, 11 studies are investigating iCRT, enrolling patients with T2-T4 M0 MIBC [32,33,34,35,36,37,38,39,40,41,42]. Screening for conference abstracts of international meetings to identify preliminary reported outcomes of these ongoing trials led to 4 abstracts. We contacted all principal investigators to ensure no additional data were available. 

### 2.1. Overview of Studies 

#### 2.1.1. Study Design

Seven studies follow a nonrandomized phase I-II design with a sample size between 30 and 80 patients to gather safety and toxicity information in order to warrant further large-scale randomized trials [31,32,33,36,39,40,41]. Five trials are comparative randomized trials (CRT vs. iCRT) based on the results of ICI use before RC and the preliminary results of ongoing phase II trials [34,35,37,38,42]. All studies include T2-T4 M0 MIBC patients (Table 1). Two studies also include patients with lymph node metastases; the CRIMI study allows lymph node metastases below the common iliac trunc (cN1), and the INSPIRE study only recruits patients N+ patients [32,35]. Generally, eligibility criteria are WHO performance status 0–2, predominantly (>50%) urothelial histology, and the absence of multifocal carcinoma in situ (CIS) in the bladder. Prior intravesical therapy with chemotherapeutics or Bacillus Calmette–Guérin (BCG) is allowed. The minimally required kidney function varies per study depending on the choice of CT.

#### 2.1.2. Study Outcomes

All studies are designed to obtain safety and toxicity data, either as part of dose-finding or to evaluate the toxicity of new iCRT combinations. Secondary outcomes are displayed in Table 1. An additional important secondary outcome in all these studies is bladder intact event-free survival (BI-EFS). 

Due to the nature of the treatment, the availability of tumor tissue to perform translational exploratory analyses is limited. However, most studies collect blood samples at different time points or compare pretreatment tumor material with bladder tissue biopsies in cases of recurrence following study treatment.

#### 2.1.3. Treatment Schedules

Different iCRT schedules and combinations are under investigation (Table 1). All studies require patients to have undergone a maximally radical TURBT, in line with current guidelines for trimodality treatment (TMT) [4]. Four studies adhere to a fixed chemotherapeutic regimen based on radiosensitizers or cisplatinum [31,32,33,39], while others offer a choice between cisplatinum, 5-FU + MMC or gemcitabine regimens (34,38,42), or an even broader choice [35,36,37,40,41]. Patients treated in the INSPIRE study receive 3 courses of induction CT, and those without progression are randomized for CRT or iCRT. 

In all studies, radiotherapy schedules consist of external beam radiation therapy (EBRT) where the whole bladder is radiated with or without a tumor boost. In most studies, adjacent lymph nodes can be included in the treatment field. Hypofractionation schedules are usually permitted. Applied doses to the bladder tumor vary from 50 Gy in 4 weeks to 64 Gy in 6.5 weeks.

Employed ICI regimens include monotherapy of anti-PD-1 (nivolumab or pembrolizumab), anti-PD-L1 (durvalumab or atezolizumab), or combined anti-PD-1 and anti-CTLA-4 (nivolumab and ipilimumab). The frequency of ICI infusions varies from once every 3 weeks to once every 6 weeks. The timing of ICI administration differs between studies. Three options are being investigated: concurrent to CRT [33,37,39], adjuvant up to one year in case of clinical response to CRT [36,38,40,41] and concurrent followed by adjuvant up to one year after CRT [31,32,34,35,42] (Figure 1). Adjuvant use of ICI is either optional for all included patients or those who achieved cystoscopically confirmed complete response following CRT. When used concurrently, ICI is usually given on the first day of CRT for a total of 4 infusions followed by adjuvant ICI depending on study design. In one study, the first round of ICI is given 2–3 weeks prior to maximal TURBT, followed by concurrent iCRT [39].

### 2.2. Reported Outcomes 

Thus far, one study has published peer-reviewed results on 8 patients treated with iCRT [31]. Four ongoing trials have presented preliminary results on 20, 10, 54, and 73 patients, respectively [32,33,39,42]. 

Marcq et al. intended to include 25 patients in a phase I trial concurrent iCRT with durvalumab (PD-L1) [31]. However, the dose of durvalumab was lowered from 1200 mg to 840 mg due to ≥grade 3 toxicity in 3 of 5 patients (2 of which were dose limiting). With the lower dose, durvalumab toxicity remained and the study was terminated after inclusion of 8 subjects. 

In the CRIMI study, patients are treated with CRT and three dose-escalating cohorts: nivolumab monotherapy, nivolumab 3 mg/kg + ipilimumab 1 mg/kg, or nivolumab 1 mg/kg + ipilimumab 3 mg/kg [32]. Preliminary results on the safety and early outcome of the nivolumab monotherapy and nivolumab 3 mg/kg + ipilimumab 1 mg/kg cohort have been reported. Patients in the phase II ANZUP-1502 and 15-00220 trials receive concurrent iCRT with pembrolizumab 200 mg (PD-1) [33,39]. Preliminary results of the ANZUP 1502 are available for the first 10 out of 30 included patients. In the 15-00220 trials, the enrollment of all 54 patients has been completed. In the phase III randomized comparative INTACT trial, patients receive either CRT or iCRT with atezolizumab (PD-L1) [42]. From this trial, toxicity data of the first 73 patients are available.

#### 2.2.1. Patient Characteristics and Outcomes

The median age of participants varies from 67 and 74 years (Table 2.). Depending on the treatment regimen, 50–100% of patients completed full study treatment (i.e., no missed or reduced doses of CT, RT or ICI). Notably, in Marcq et al. [31] all 8 patients completed CRT and had at least 2 ICI infusions. However, in 3 patients ICI treatment had to be discontinued due to treatment-related toxicity. In the trials with a single ICI agent approximately 80% of patients completed full treatment compared to 50% in the nivolumab + ipilimumab ICI combination cohort of the CRIMI study (mostly reduction of capecitabine or withholding of an ICI infusion). Grade ≥3 adverse events (AEs) were common in all studies (10 and 62%). Notably, in the only comparative trial (INTACT) iCRT led to more serious AE than CRT, 62% vs. 30%. Early treatment outcome data reported complete responses in 90% of treated subjects and 1-year DFS, MFS and OS ranging between 77% and 100%. 

#### 2.2.2. Treatment Toxicity

Table 3 shows the reported immune-mediated toxicity. Overall, any grade toxicity was seen in around 10% of patients. Marcq et al. reported a much higher grade 3 immune-mediated toxicity in 5 out of 8 patients [31]. Low-grade urinary tract AEs were seen between 10% and 63% and mainly consisted of urinary tract infections (UTI) or acute kidney injury (AKI). In the INTACT trial, grade 3 urinary tract AEs occurred in 25% of patients in the iCRT cohort vs. 3% of CRT-only patients [42]. The occurrence of any hematological AE during iCRT was common at around 20–30% in all studies. It is unknown if hematological AEs were clinically significant and required any dose reductions. Gastrointestinal toxicity varied between studies with no occurrence in the ANZUP-1520 and INTACT trials and up to 50% in Marcq et al. [31,33,42]. In the latter study, the main toxicity was (biopsy proven) colitis, occurring in 3 patients out of 8 patients. Overall, colitis was the most reported gastrointestinal toxicity. In some cases, it was deemed to be related to CRT rather than to ICI. Two deaths occurred during iCRT study treatments: one in the nivolumab 3 mg/kg + ipilimumab 1 mg/kg cohort of the CRIMI study and one in the 15-00220 [32,39]. Both were deemed unrelated to ICI use.

## 3. Discussion

This review provides an overview of studies using ICI and CRT in a combined iCRT regimen for bladder-sparing treatment of MIBC patients. We found a total of 12 iCRT studies. Ten studies are actively recruiting, of which four have reported preliminary data during international conferences. Most studies combine CRT with anti-PD-1 or anti-PD-L1 monotherapy, and one combines anti-PD-1 and anti-CTLA-4 with CRT. Apart from Marcq et al. [31], most studies reported toxicity profiles that are acceptable in a curative treatment setting. Early treatment outcomes of this new treatment modality, such as response rate, 1-year DFS, and MFS, seem promising.

**Figure 2:**
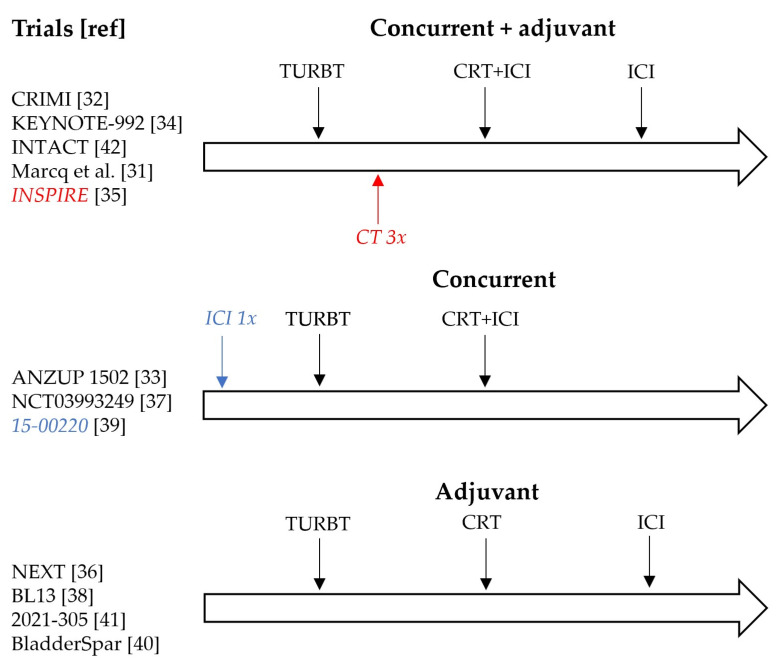
Overview of different CRT + ICI schedules under investigation.

The trials in this review schedule the ICI administrations either adjuvant to CRT, concurrent to CRT, or a combination. Within these three scheduling options, there is a remarkable variation in the timing of the TMT components (Figure 2). There are different rationales for these variations based either on experience with iCRT in other tumor types, on perioperative ICI in the context of RC, or on ICI use in the treatment of mUC patients.

Adjuvant nivolumab use has shown improved DFS versus placebo for high-risk nonmetastatic MIBC urothelial carcinoma patients after RC and has obtained FDA approval, creating a rationale for adjuvant administration of PD-1 [43]. Additionally, adjuvant ICI after CRT led to improved OS compared to placebo in NSCLC [26]. 

Eight trials apply concurrent administration of ICI with CRT. Van den Ende et al. showed the feasibility of concurrent ICI use with CRT in a neoadjuvant setting before resectioning of esophageal carcinoma [44]. Furthermore, a recent nonrandomized phase II trial of pembrolizumab concurrent to CRT in patients with locally advanced stage III NSCLC demonstrated objective response rates of 70% [45]. Although toxicity in this regime was relatively high, with 58% of patients experiencing any grade 3–5 AEs leading to treatment discontinuation in 26%, this seems acceptable considering the historical response rates of CRT between 35.9 and 54.5% [46,47,48]. 

With the introduction of the iCRT treatment modality, there are some limitations to take into account. First, several CRT regimens are being combined with ICI, as the optimal CRT treatment regimen for MIBC is still undecided. This may complicate future comparisons. Recently, a meta-analysis of patients treated in the BCON and BC2001 trials confirmed the superiority of hypofractionation with 55 Gy in 20 fractions over 64 Gy in 32 fractions [49]. Despite these data, only four iCRT studies use a hypofractionated schedule, of which two use the exact dose of 55 Gy. In addition, four studies chose to irradiate pelvic lymph nodes, which increases toxicity, and available data do not suggest improved outcomes over whole-bladder-only irradiation [50,51]. 

Chemotherapy combinations with cisplatinum are considered most effective for urothelial carcinoma [7]. Cisplatinum, followed by gemcitabine and mitomycin C with fluorouracil, is the most used chemosensitizer included in this review. Results from the KEYNOTE-361 trial and IMvigor130 did not reveal a synergistic effect for the combination of CT with ICI in metastatic urothelial carcinoma, and toxicity might be the primary factor to consider when deciding upon which radiosensitizing CT should be used [52,53]. Cisplatinum is known to cause substantial toxicity; therefore, other schedules have been proposed. One study uses the oral prodrug capecitabine instead of 5 FU. However, limited data are available on the clinical use of this combination in bladder cancer [54]. 

A second limitation is the lack of standardized reporting of iCRT treatment outcomes. A standardized response evaluation after iCRT has not yet been defined, despite the use of regular follow-up cystoscopies with or without tumor site biopsies and CT scans. The response criteria in the solid tumors (RECIST) workgroup proposed an ICI updated version of RECIST, but these guidelines focused on advanced metastatic disease, and its role in iCRT follow-up is unclear [55]. Accurate cystoscopic response evaluation of the bladder following this iCRT might prove challenging. Four studies explicitly describe routine bladder biopsies to objectify response rates (ref). However, the extent of the aforementioned biopsies is not reported. When performed, biopsies, as well as TURBT-specimens, are subject to sampling bias and can be difficult to interpret for the pathologist following extensive multimodality therapy [56]. As can be with an organ-sparing treatment, some iCRT studies in this review adhere to endpoints related to bladder preservation. However, this definition varies between studies. Some include NMIBC recurrences, while others focus only on MIBC recurrences, locoregional metastases, or salvage cystectomy rates. This lack of standardized outcome reporting complicates future comparisons of the effectiveness of different iCRT regimens. 

Thirdly, the studies mentioned in this review are primarily phase I–II trials, which can lead to a selection bias towards patients with a relatively high performance status. It is unclear whether results can be extrapolated to the majority of bladder cancer patients undergoing bladder-sparing treatment, which currently are often older, less fit, and more comorbid [20].

Lastly, biomarker-based identification of potential nonresponders can be a future challenge for iCRT treatments. Several studies report PD-L1 expression as a prognostic factor for response to anti-PD-(L)1. Therefore, in some cases, the derivative combined positive score (CPS) is required as a predictive biomarker [57]. Furthermore, Necchi et al. identified an RNA-based immune signature associated with complete remission after pembrolizumab induction therapy preceding RC. Additionally, these patients had improved PFS [58]. In contrast to studies combining ICI with RC, where tissue after ICI is abundant, the availability of tissue following bladder-sparing iCRT is limited. This will complicate research into biomarkers for response and immune evasion of tumors treated with iCRT. 

Phase 2 results on ICI use as induction therapy prior to RC show a pCR in up to 45% of patients [13,14,15,16]. Comparative trials of ICI with or without neoadjuvant CT prior to RC are already underway [59]. Interestingly, one of the iCRT studies included in this review administers ICI in an induction setting prior to TURBT [39]. However, only a single infusion of ICI is given prior to the full treatment instead of a series of ICI infusions as in several RC studies. Despite the earlier mentioned synergy, CRT might also be applied as a consolidative treatment following upfront ICI in MIBC. This might prove a solution if the concurrent use of iCRT leads to intolerable acute toxicity. However, as immune-related adverse events (irAE) often occur 1–2 months after ICI administration, the risk that CRT has to be postponed due to irAE increases. 

In summary, several trials are investigating ICI combinations with CRT, and early peer-reviewed results are to be expected in early 2022. Based on preliminary results, the future of bladder preservation therapy for MIBC could be promising. The results of the two phase 3 trials will hopefully answer the question on the additional benefit of ICI to this multimodality approach. 

## 4. Conclusions

There is a strong rationale for combining ICI with existing bladder-sparing CRT, which could reshape the current treatment landscape of MIBC. Multiple studies investigating different combinations are ongoing. Preliminary results on toxicity and early treatment outcomes are encouraging but not yet definitive. Peer-reviewed published results are expected in the coming years.

## Figures and Tables

**Table 1:** General overview of currently enrolling studies.

NCT (Title), Ref	Phase	N	ICI (CP)	Chemotherapy	Radiotherapy	Concurrent/Adjuvant	Primary Endpoint	Secondary Endpoints
NCT03620435 (Marcq et al.) [31]	I	8	Atezolizumab (PD-L1)	Gem	20 × 2.5 Gy and 20 × 2 pelvic nodes IMRT	Concurrent + adjuvant 9 m	Safety/toxicity	RR, OS, QoL
NCT03844256 (CRIMI) [32]	Ib/II	30–50	Nivolumab (PD-1) +/− Ipilimumab (CTLA-4)	MMC + Cape	20 × 2 Gy whole bladder, tumor boost 20 × 0.75 Gy	Concurrent + 1 y adjuvant nivolumab	Safety/toxicity	DFS, OS
NCT03171025 (NEXT) [36]	II	28	Nivolumab (PD-1)	Radiosensitizing NOS	NR	Adjuvant 1 y	Failure free survival	BI-EFS, AE Cystectomy rate QoL
NCT03993249 [37]	II	78	Nivolumab (PD-1)	SoC	SoC	Concurrent	Locoregional control rate	AE, RFS, OS, QoL
NCT02662062 (ANZUP 1502) [33]	II	30	Pembrolizumab (PD-1)	Cis	32 × 2 Gy	Concurrent	Safety/toxicity	RR, MFS, Cystectomy rate
NCT02621151 (15-00220) [39]	II	54	Pembrolizumab (PD-1)	Gem	20 × 2.6 Gy	Concurrent	BI-DFS	AE, RR, MFS, OS
NCT04216290 (INSPIRE) [35]	II	114	Durvalumab (PD-L1)	Gem *or* Gem + Carbo *or* Gem + Cis *or* MVAC	6.5–8 weeks NOS	Concurrent + 9 m adjuvant	Complete response rate	BI-EFS, Cystectomy rate, PFS, MFS, CSS, OS
NCT03768570 (BL13) [38]	II	190	Durvalumab (PD-L1)	Cis *or* 5-FU + MMC *or* Gem	Bladder: 32 × 2 Gy *or* 20 × 2.5 Gy. Pelvis: 45–46 Gy + 17–20 Gy bladder	Adjuvant 1 y	DFS	BI-EFS, locoregional control, MFS
NCT03697850 (BladderSpar) [40]	II	77	Atezolizumab (PD-L1)	SoC	≥60 Gy	Adjuvant 1 y	DFS	Local control, AE, DFS, OS, QoL
NCT04241185 (Keynote-992) [34]	III	636	Pembrolizumab (PD-1)	Cis *or* 5-FU + MMC *or* Gem	32 × 2 Gy +/− Nodes or 20 × 2.75 Gy	Concurrent + 1 y adjuvant	BI-EFS	AE, OS, MFS. NMIBC recurrence, QoL
NCT05072600 (2021-305) [41]	III	54	Pembrolizumab (PD-1)	SoC	SOC	Adjuvant 1 y	PFS	NR
NCT03775265 (INTACT) [42]	III	475	Atezolizumab (PD-L1)	Cis *or* 5-FU + MMC *or* Gem	Bladder *or* Pelvis	Concurrent + 6 m adjuvant	BI-EFS	AE, OS, QoL

NCT: national clinical trial number, ICI: immune checkpoint inhibitors, DFS: disease-free survival, OS: overall survival, RR: response rate, MFS: metastatic free survival, AE: adverse events, BI-EFS: bladder intact event-free survival, NMIBC: non-muscle-invasive bladder cancer, QoL: quality of life, PFS: progression-free survival, CSS: cancer-specific survival, CP: Checkpoint targeted, MMC: mitomycin C, Cape: capecitabine, Cis: cisplatinum, 5-FU: fluorouracil, Carbo: carboplatin, MVAC: methotrexate vinblastine, adriamycin, cisplatinum, SoC: standard of care, Gy: gray, NOS: not otherwise specified, NR: not reported, IMRT: intensity-modulated radiation therapy, PD-1: programmed death 1, PD-L1: programmed death-ligand 1, CTLA-4: cytotoxic T lymphocyte antigen-4, Gem: gemcitabine.

**Table 2:** Preliminary results of studies on ICI + CRT.

NCT (Title), Ref	*N* (Total)	Age y (Median)	Completed Full Therapy	Gr ≥ 3 AEs	Complete Response	Additional Outcome
NCT03620435 (Marcq et al.) [31]	8	68	63%	62.5% (5/8)	NR	NA
NCT03844256 (CRIMI) [32]	Nivo: 10 Nivo/ipi 10 (50)	Nivo: 68 Nivo/ipi: 70	nivo: 100% nivo/ipi: 50%	Nivo: 10% (1/10) Nivo/ipi: 30% (3/10)	NR	Nivo (1 year): DFS 100% OS 100%
NCT02662062 (ANZUP 1502) [33]	10 (30)	NR	80% *(1 CT, 1 ICI)*	40% (4/10)	9/10 (at 24 weeks)	24-week MFS 90%
NCT02621151 (15-00220) [39]	SC: 6 EC: 48 (54)	SC 67 EC 74	88% *(1 CRT, 2 CT, 4 ICI)*	31% (17/54)	±87% (at 12 weeks)	1-year eBI-DFS 77% (95% CI: 0.60–0.87). 1-year MFS 85% (95% CI 0.71–0.93)
NCT03775265 (INTACT) [42]	73 (475)	NR	NR	iCRT 62% (23/37) CRT 30% (11/35)	NR	NA

NR: not reported, eBI-DFS: estimated bladder intact disease-free survival, CI; confidence interval, Gr; grade, CR: complete response, DFS: disease-free survival, OS: overall survival, MFS: metastatic free survival, SC: safety cohort, EC: extension cohort, ICI: checkpoint inhibition, CRT: chemoradiotherapy, NCT: national clinical trial number, NA: not applicable.

**Table 3:** Reported immune-mediated, urinary tract, and hematological adverse events.

NCT (Title), Ref	Immune AEs Gr ≤ 2	Immune AEs Gr ≥ 3	Urinary AEs Gr ≤ 2	Urinary AEs Gr ≥ 3	Hematological AEs Gr ≤ 2	Hematological AEs Gr ≥ 3
03620435 (Marcq et al.) [31]	25% (2/8)	63% *(5/8 Colitis, lymphopenia)*	63% *(5/8 dysuria)*	0%	*-Anemia 25% (2/8) -Lymphopenia 25% (2/8) -Neutropenia 25% (2/8)*	*-Neutropenia 13% (1/8) -Lymphopenia 13% (1/8)*
03844256 (CRIMI) [32]	Nivo: 10% *(1/10 hepatitis*) Nivo3/ipi1: 20% *(2/10 colitis, pancreatitis)*	Nivo: 0% Nivo3/ipi1: 10% *(1/10 colitis)*	Nivo: 10% *(1/10 AKI)* Nivo3/Ipi1: 40% *(4/10 UTI, AKI, Urgency)*	Nivo: 0% Nivo3/Ipi1: 10% *(1/10 UTI)*	Nivo: 0% Nivo3/Ipi1: 20% *(2/10 anemia, thrombocytopenia)*	Nivo: 0% Nivo3/Ipi1: 20% *(2/10 anemia, thrombocytopenia)*
02662062 (ANZUP 1502) [33]	10% *(1/10 Nephritis)*	0%	NR	0%	NR	NR
02621151 (15-00220) [39]	NR	7% *(4/54 GI)*	NR	12% *(6/54 UTI, obstruction)*	NR	4% *(2/54 neutropenia, thrombocytopenia)*
03775265 (INTACT) [42]	NR	0%	iCRT: 9% *(3/37 UTI)* CRT: 9% *(3/36 AKI 1, UTI 2)*	iCRT: 25% *(9/37 AKI 2, UTI 7)* CRT: 3% *(1/36 UTI)*	iCRT: *-Anemia 41% (15/37)* *-Lymphopenia 3% (1/37)* *-Neutropenia: 20% (7/37)* *-Leukopenia 22% (8/37)* CRT: *-Anemia 33% (12/36)* *-Lymphopenia 0%* *-Neutropenia 11% (4/36)* *- Leukopenia 14% (5/36)*	iCRT: *-Anemia 4% (11/37)* *-Lymphopenia 16% (6//37)* *-Neutropenia: 8% (3/37)* *-Leukopenia 19% (7/37)* CRT: *-Anemia 3% (1/36)* *-Lymphopenia 17% (6/36)* *-Neutropenia 8% (3/36)* *-Leukopenia 8% (3/36)*

NCT: national clinical trial number, AE: adverse event, UTI: urinary tract infection, AKI: acute kidney injury, GI: gastrointestinal, NR: not reported.
